# Physiological, canopy, and yield responses of quinoa to irrigation and nitrogen management in the U.S. Midwest

**DOI:** 10.3389/fpls.2026.1780275

**Published:** 2026-03-12

**Authors:** Fatema Tuj Johora, Grato Ndunguru, Safiullah Pathan, Xukai Zhang, Nur Un Nesa, Addissu G. Ayele

**Affiliations:** Department of Agriculture and Environmental Sciences, Lincoln University of Missouri, Jefferson City, MO, United States

**Keywords:** gas exchange, grain filling, irrigation management, NDVI, nitrogen-use efficiency, quinoa

## Abstract

Quinoa (*Chenopodium quinoa* Willd.) exhibits substantial physiological plasticity under water and nitrogen (N) stress; however, the mechanistic integration of stomatal regulation, canopy development, and agronomic efficiency of nitrogen (AE_n_) under humid temperate field conditions remains poorly understood. This study mechanistically evaluated the effects of irrigation regime and N rate on leaf gas exchange, canopy vigor, AE_n_, and grain yield during the 2024 and 2025 growing seasons in the U.S. Midwest. Two genotypes were tested under four irrigation regimes (full, progressive, deficit, and extreme deficit) and three N rates (0, 75, and 150 kg N ha^−1^) using a split-split plot design. The results indicated that the high-yielding genotype consistently produced higher grain yield across treatments, whereas the low-yielding genotype consistently produced lower grain yield; however, both genotypes responded similarly to irrigation and N application, with no significant genotype × management interactions. Irrigation primarily regulated gas exchange during early growth and flowering, whereas nitrogen status and irrigation × N interactions governed physiological performance during grain filling. Increasing N enhanced net photosynthesis, stomatal conductance, and NDVI at peak canopy development, but these increases did not translate into proportional yield gains at the highest N rate. Grain yield at 150 kg N ha^−1^ under extreme deficit irrigation was comparable to that at 75 kg N ha^−1^ under progressive irrigation, indicating strong water–nitrogen trade-offs. Agronomic efficiency of nitrogen differed significantly among irrigation regimes, whereas N rate and genotype had no significant effects, and AE_n_ declined with increasing N input. Overall, moderate irrigation combined with 75 kg N ha^−1^ was associated with improved physiological performance, canopy vigor, and favorable grain-yield and agronomic efficiency nitrogen responses, highlighting grain filling as the primary physiological control point under humid temperate conditions.

## 1 Introduction

Quinoa (*Chenopodium quinoa* Willd.) is a nutrient-dense, gluten-free pseudocereal recognized internationally for its superior nutritional composition, including elevated protein content, a complete essential amino acid profile, and significant nutritional value across all edible tissues, including grains, green leaves, and inflorescences (Pathan et al., 2024). Demonstrating tolerance to abiotic stresses such as drought, salinity, and extreme temperatures, quinoa is increasingly vital for climate-resilient food systems (Bazile et al., 2016; Hinojosa et al., 2018). Although quinoa originated in the Andean region of South America, its cultivation has expanded to more than 120 countries, driven by demand for alternative crops that can sustain productivity under fluctuating environmental conditions.

Quinoa yield is influenced by local climate, soil, and, particularly, agronomic practices. Therefore, maximizing water and nutrient use is crucial, especially in non-traditional regions where environmental conditions differ from those of quinoa’s origin. In a humid temperate environment such as the U.S. Midwest, the crop is exposed to variable rainfall, drought episodes, high humidity, and uneven soil fertility. These factors create complex interactions among water supply, nitrogen dynamics, and plant physiology, underscoring the need for region-specific management strategies. Irrigation management is a critical practice for maximizing quinoa growth, physiological function, and yield, particularly in environments with variable precipitation. Studies in arid and semi-arid regions have shown that deficit irrigation regimes can sustain quinoa productivity while enhancing water-use efficiency (Geerts et al., 2008; Valdivia-Cea et al., 2021; Çolak et al., 2021; El-Harty et al., 2023). These findings suggest that quinoa tolerates moderate water deficits through effective stomatal regulation, thereby maintaining photosynthetic activity and intrinsic water-use efficiency. In contrast, prolonged or severe drought typically constrains stomatal conductance, reduces carbon assimilation, and ultimately limits biomass accumulation and grain yield. Genotypic variation in responses to water availability further highlights the role of physiological plasticity in quinoa drought adaptation. Evidence from temperate environments similarly supports quinoa’s capacity to adjust to contrasting water regimes. Pathan et al. (2023) evaluated multiple quinoa genotypes under irrigated, rainfed, and drought-stress conditions and reported significant genotype × water-regime interactions for grain yield and morphological traits. Under drought-stress conditions, quinoa often produced yields comparable to, and in some cases better than, those under irrigated and rainfed treatments (Pathan et al., 2023), demonstrating its ability to maintain productivity under reduced water supply.

Physiological studies offer insights into how quinoa tolerates water stress. Drought conditions usually reduce stomatal conductance, transpiration, and photosynthesis. However, intrinsic water-use efficiency often stays high (Flexas et al., 2008; Chaves et al., 2009; Hinojosa et al., 2018). Gas-exchange traits, such as photosynthetic rate (A), stomatal conductance (gsw), transpiration (E), and intercellular CO_2_ (Ci), are influenced by soil moisture. These traits are reliable indicators of crop performance under water-limited conditions. Recent studies indicate that abiotic stress regulates stomatal conductance, which jointly limits CO_2_ diffusion and photosynthesis in C_3_ crops (Li et al., 2022). Still, responses to water alone do not fully explain yield differences, as nitrogen availability interacts with water to affect carbon assimilation and biomass allocation.

Nitrogen is a key limiting nutrient for quinoa, influencing canopy development, chlorophyll levels, photosynthetic capacity, and yield. Studies generally report positive responses to increased nitrogen applications. However, over-application can increase the risk of plant lodging, particularly in quinoa under favorable moisture conditions (Razzaghi et al., 2012; Hawkesford, 2014; Pradhan et al., 2025). The impact of nitrogen fertilization depends on water supply, as adequate soil moisture is necessary for nitrogen uptake and use. Therefore, interactions between irrigation and nitrogen are critical for quinoa yield and nitrogen-use efficiency, with outcomes varying across environments and management practices (Alvar-Beltrán et al., 2020; Wang et al., 2021; Bahrami et al., 2022). Recent advances in crop phenotyping enable new approaches to assessing crop responses to irrigation and nitrogen. Optical vegetation indices, such as the normalized difference vegetation index (NDVI), enable rapid, non-destructive measurement of canopy structure, development, and vigor. NDVI has been successfully used in quinoa and other crops (Alvar-Beltrán et al., 2020; Mitra et al., 2023). NDVI trends during peak growth can supplement physiological and nitrogen-use efficiency data, improving understanding of how management affects crop performance.

Despite extensive global research on quinoa, little is known about the combined effects of irrigation and nitrogen management in humid temperate environments, such as those in the U.S. Midwest (Jacobsen et al., 2003). Most U.S. research focuses on western regions. Few studies have examined how physiological processes, canopy dynamics, yield, and nitrogen-use efficiency are coordinated under Midwestern conditions. Unlike arid and semi-arid production systems, where water availability is the primary limiting factor for quinoa, humid temperate environments such as Missouri present distinct challenges driven by interactions among high atmospheric humidity, episodic rainfall, and soil physical constraints. Missouri soils are commonly clay-influenced and have moderate to high water-holding capacity, conditions that can restrict oxygen diffusion in the root zone during heavy watering or intense rainfall. Under such conditions, high soil moisture may exacerbate root-zone hypoxia, reduce nitrogen uptake efficiency, and increase disease pressure, potentially offsetting the benefits of increased water supply (Hinojosa et al., 2018). Consequently, deficit irrigation in humid temperate systems may improve root-zone aeration and nitrogen-use efficiency rather than induce classical drought stress, as reported for quinoa and other crops grown under high soil moisture conditions (Razzaghi et al., 2012; Hinojosa et al., 2019). However, the physiological and agronomic mechanisms governing these responses remain poorly quantified for quinoa under Midwestern field conditions. In contrast to prior studies that evaluate irrigation or nitrogen management separately, this work integrates gas exchange, drone-based NDVI, and agronomic nitrogen efficiency. The aim is to identify stage-specific constraints on quinoa productivity in a humid temperate environment. The objectives of this study are to (i) examine nitrogen-driven responses under varying irrigation conditions in the Midwest, (ii) assess morphological and physiological responses of quinoa using both manual and high-throughput aerial phenotyping methods, and (iii) evaluate the agronomic efficiency of nitrogen (AE_n_) under combined irrigation and nitrogen management. The hypothesis is that quinoa requires relatively low or moderate soil moisture and intermediate nitrogen rates to maximize resource use and boost productivity in the U.S. Midwest.

## 2 Materials and methods

### 2.1 Study location and planting materials

The study was conducted during the summers of 2024 and 2025 at the George Washington Carver Farm of Lincoln University in Jefferson City, Missouri, USA (38.32° N, 92.80° W; 171 meters above sea level), with a pH of 6.5-6.8. Quinoa was planted on 3 June and harvested on 5 September 2024. In 2025, planting occurred on 28 May and harvesting on 4 September. Two quinoa (*Chenopodium quinoa* Willd.) genotypes with contrasting yield potential, PI698769 (formerly Ames 13746; origin: New Mexico, USA) and PI614885 (origin: Chile), were used in this study. These genotypes were selected from a preliminary evaluation of ten quinoa accessions based on prior assessments of grain yield and agronomic performance under irrigated, rainfed, and water-stress conditions (Pathan et al., 2023). The purpose of selecting genotypes with contrasting yield capacity was not to compare genotypic yield performance per se, but to evaluate whether quinoa genotypes differing in baseline yield potential exhibit similar or divergent physiological, canopy, and yield responses to irrigation and nitrogen management. Seed material was obtained from the USDA-ARS Germplasm Resources Information Network (GRIN; North Central Regional Plant Introduction Station, Ames, IA, USA) and multiplied at Lincoln University of Missouri (Jefferson City, MO, USA) prior to field experimentation.

**Figure 1:**
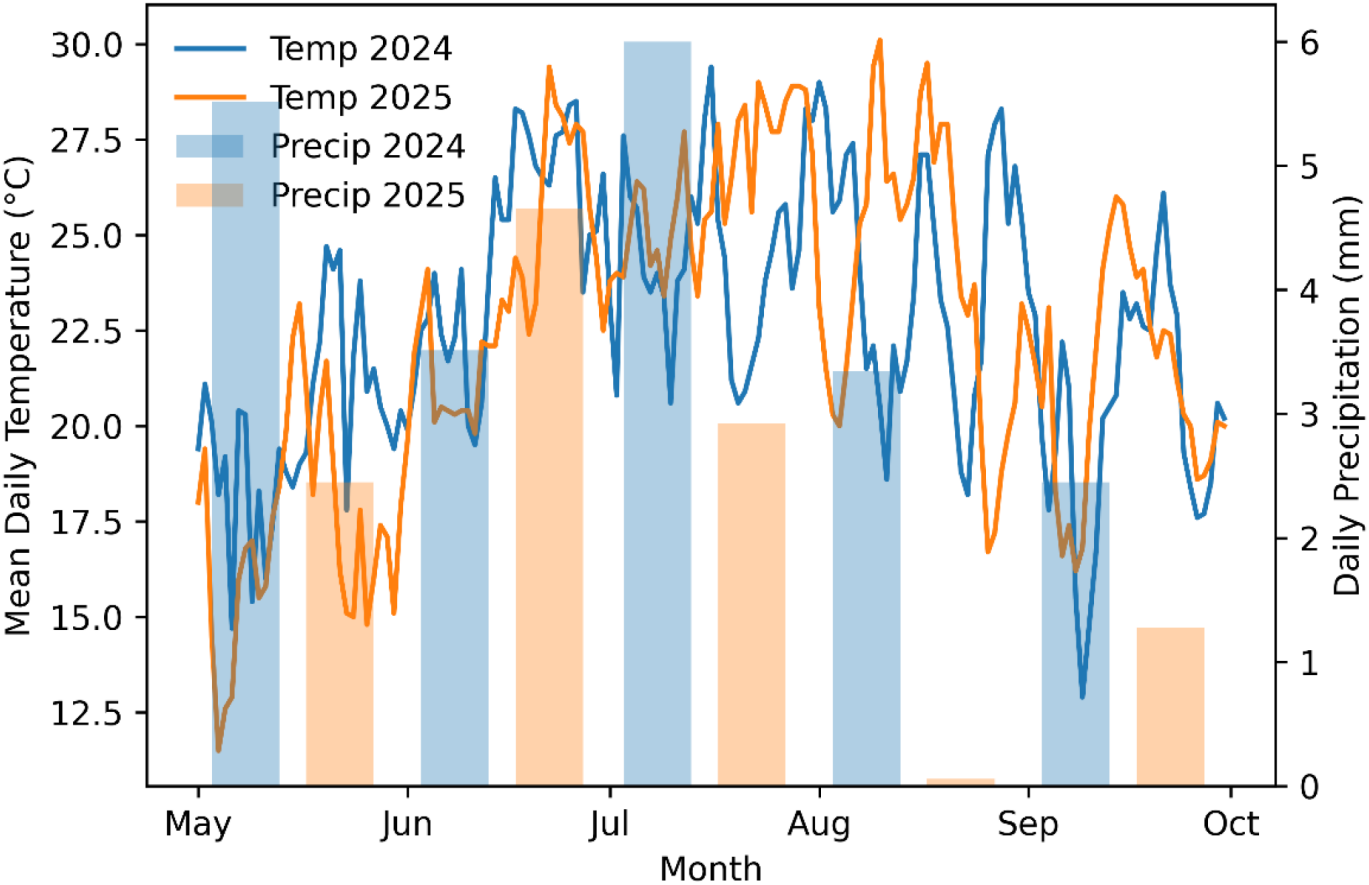
Mean daily air temperature (solid lines) and daily precipitation (bars) recorded during the quinoa growing seasons (May–September) in 2024 and 2025 at the George Washington Carver Farm, Jefferson City, Missouri.

Daily mean air temperature and precipitation patterns during the growing seasons (May–September) of 2024 and 2025 at Carver Farm, Jefferson City, Missouri, are shown in **Figure 1**. Daily mean temperature is shown as continuous lines for each year, with calendar months overlaid to facilitate comparison between years (**Figure 1**). Precipitation is shown as side-by-side bars (**Figure 1**) for each month, representing the aggregated daily precipitation for each year. Overall, temperature trends were similar across years, with higher midsummer temperatures in July and August. However, precipitation distribution varied significantly, with 2024 receiving more rainfall in the early and midseason than 2025, especially in August.

**Figure 2:**
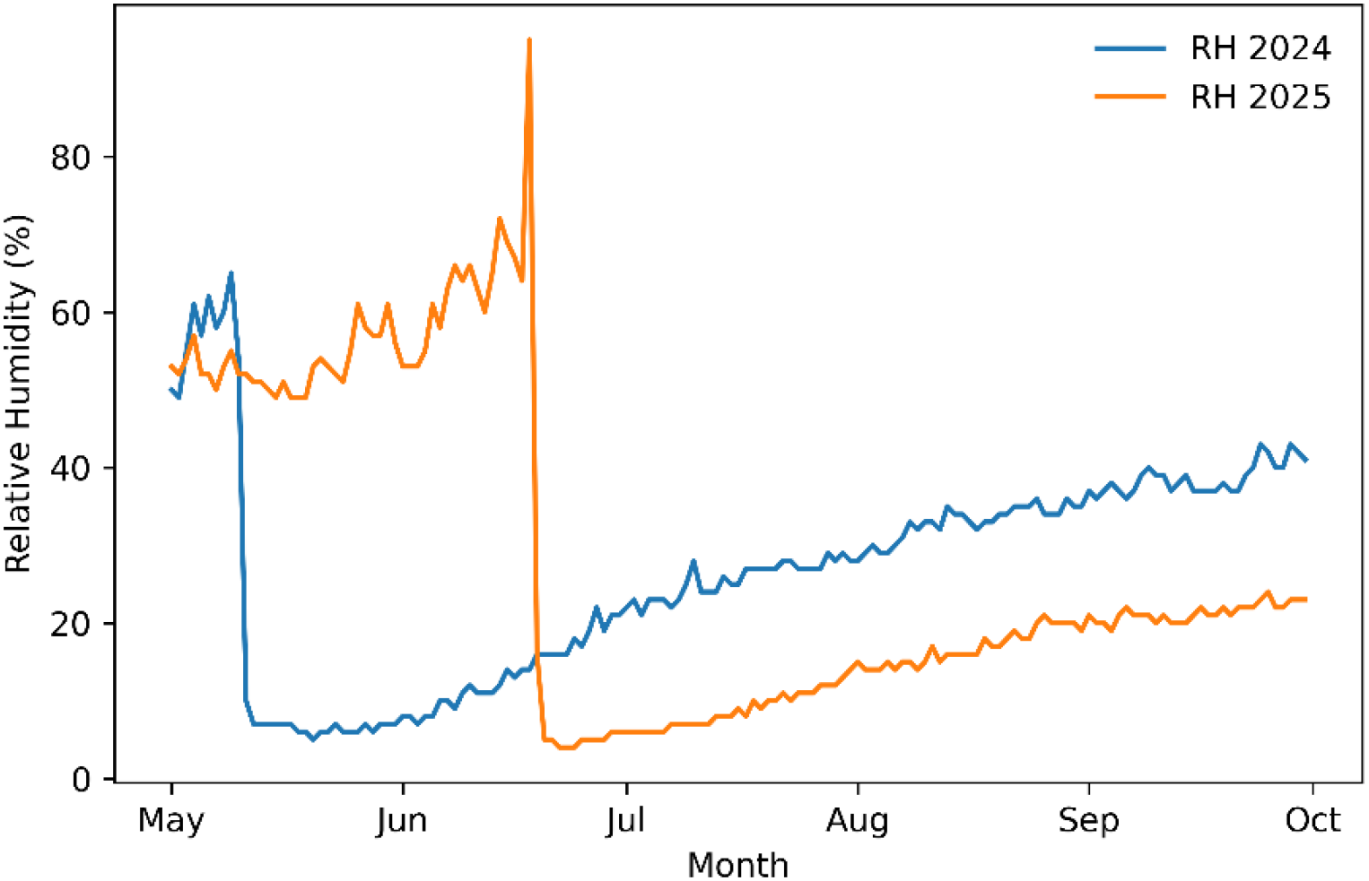
Daily mean relative humidity (RH) during the growing seasons (May–September) of 2024 and 2025 at George Washington Carver Farm, Jefferson City, Missouri.

Relative humidity peaked in July, coinciding with the most significant disease infestation observed since the start of quinoa research at Lincoln University. Although disease pressure can be influenced by many factors, such as heavy nitrogen availability (**Table 1**), the high humidity (**Figure 2**) during this period likely created conditions favorable for pathogen growth.

**Table 1:** Analysis of variance (ANOVA) results showing the effects of variety (Var), irrigation (Irr), nitrogen fertilizer rate (Nitro.), and their interactions on gas-exchange parameters at bud initiation, grain filling, and flowering stages.

Source	Bud initiation				Grain filling				Flowering			
	gsw	E	A	Ci	gsw	E	A	Ci	gsw	E	A	Ci
Var	0.15	0.05	0.05	0.05	0.05	0.05	0.14	0.58	0.15	0.15	0.23	0.73
Irr	0.05	0.01	0.01	0.05	0.49	0.14	0.01	0.67	0.001	0.001	0.30	0.97
Nitro.	0.08	0.16	0.16	0.01	0.01	0.01	0.001	0.17	0.58	0.58	0.05	0.24
Var × Irr	0.05	0.06	0.06	0.05	0.26	0.21	0.26	0.01	0.50	0.50	0.69	0.54
Var × Nitro.	0.39	0.48	0.48	0.07	0.64	0.76	0.83	0.73	0.83	0.83	0.21	0.32
Irr × Nitro.	0.36	0.90	0.90	0.19	0.01	0.01	0.01	0.01	0.86	0.86	0.34	0.14
Var × Irr × Nitro.	0.35	0.27	0.27	0.50	0.24	0.19	0.55	0.16	1.00	1.00	0.98	0.29

Significance levels are indicated as P < 0.05, P < 0.01, and P < 0.001 . Values are P-values for main effects and interactions on stomatal conductance (gsw), transpiration rate (E), net photosynthetic rate (A), and intercellular CO_2_ concentration (Ci).

### 2.2 Experimental design

The experiment was conducted as a split–split plot in a randomized complete block design with three replications, in which variety was assigned to main plots, irrigation regime to subplots, and nitrogen rate to sub-subplots. This design was adopted because irrigation treatments required larger experimental units and fixed infrastructure, whereas nitrogen could be applied with greater precision at smaller spatial scales. Each plot measured 0.91 × 3.1 m2 and was established and maintained on a raised bed, protected with plastic mulch to reduce weed pressure and promote uniform irrigation distribution. Plots were arranged in parallel rows, with adjacent rows separated by a 6-ft buffer and plots within rows by a 3-ft buffer. This buffer distance reduced canopy overlaps and minimized interference among treatments in neighboring plots.

### 2.3 Nitrogen management

Prior to nitrogen fertilizer application, soil samples were collected at 0–20 cm and 20–40 cm depths and analyzed for pH, organic matter, cation exchange capacity (CEC), extractable nutrients, and inorganic nitrogen (NO_3_^−^–N and NH_4_^+^–N) using standard laboratory procedures. Nitrogen was applied at three rates (0, 75, and 150 kg N ha^−1^) using urea as the N source. In 2024, all nitrogen was applied as a single dose 32 days after planting due to excessive rainfall during early crop establishment, whereas in 2025, nitrogen was split into two applications at 15 and 42 days after planting (DAP). Year-to-year differences in nitrogen application timing were accounted for statistically by including year as a random effect in the mixed-effects model.

### 2.4 Irrigation scheduling and application

Irrigation treatments were based on an ET_0_-derived seasonal supplemental irrigation target of 430 mm, which defined the full-irrigation regime and proportional deficit treatments: progressive irrigation (PI = 75%), deficit irrigation (DI = 50%), and extreme deficit irrigation (EDI = 25%). This target was selected because cumulative reference evapotranspiration during the quinoa growing season was similar across years (432 mm in 2024 and 429 mm in 2025), ensuring comparability of irrigation demand between seasons. The seasonal irrigation target of 430 mm served as a design benchmark to establish fixed daily irrigation rates for each treatment (**Table 2**) and does not represent total crop water demand. Irrigation was applied using a two-phase schedule. During the early vegetative phase, all plots received identical full irrigation at a mean daily rate of 4.26 mm d^−1^ to ensure uniform crop establishment and early canopy development. In 2024, this common full-irrigation phase extended from June 3 to July 14, whereas in 2025 it extended from May 28 to July 20. Differential irrigation treatments were then imposed during a late-season window, from July 15 to August 24 in 2024 and from July 21 to August 28 in 2025, with irrigation rates reduced proportionally according to treatment level (PI, DI, and EDI).

**Table 2:** Fixed seasonal irrigation depths and corresponding mean daily irrigation rates applied uniformly to quinoa during the growing season in 2024 and 2025.

Treatment	Seasonal irrigation (mm)	Mean daily irrigation (mm day^−1^)
Full	430.0	4.26
PI	322.5	3.19
DI	215.0	2.13
EDI	107.5	1.06

Full irrigation (Full) was defined as a fixed seasonal irrigation depth of 430 mm, selected based on the close agreement in cumulative reference evapotranspiration between 2024 (432 mm) and 2025 (429 mm). Deficit irrigation treatments (PI, DI, and EDI) were calculated as fixed fractions of Full (75%, 50%, and 25%, respectively). Irrigation depths were applied uniformly across the growing period in both years.

Irrigation water was delivered through a surface drip irrigation system installed under plastic mulch. Irrigation scheduling was time-controlled, and all replicate plots within each irrigation treatment were irrigated simultaneously using independent drip lines connected to a standard manifold, ensuring uniform water application within treatments. Because natural precipitation was not excluded, irrigation treatments represent fixed supplemental irrigation inputs rather than direct replacements for crop evapotranspiration demand. Daily rainfall was recorded using on-site weather observations and summed over the irrigation-managed period. To validate realized water availability under humid temperate conditions, total water input (rainfall + irrigation) for each treatment was calculated based on daily rainfall and the actual irrigation schedule and is reported in **Table 3**. While **Table 2** summarizes the ET_0_-based irrigation targets and planned daily irrigation rates that define the treatment structure, **Table 3** reports the realized irrigation applied, concurrent rainfall, and total water input during the irrigation-managed period, thereby confirming treatment separation and validating the imposed irrigation stress.

**Table 3:** Seasonal irrigation, rainfall, and total water input (rainfall + irrigation) across irrigation regimes in 2024 and 2025. Phase 1 represents the common pre-treatment irrigation period, whereas Phase 2 represents the late-season differential irrigation period.

Year	Treatment	Phase 1 irrigation (mm)	Phase 2 irrigation (mm)	Total irrigation (mm)	Total rainfall (mm)	Total water input (mm)
2024	Full	178.92	174.66	353.58	338.33	691.91
	PI	178.92	130.79	309.71	338.33	648.04
	DI	178.92	87.33	266.25	338.33	604.58
	EDI	178.92	43.46	222.38	338.33	560.71
2025	Full	230.04	166.14	396.18	236.73	632.91
	PI	230.04	124.41	354.45	236.73	591.18
	DI	230.04	83.07	313.11	236.73	549.84
	EDI	230.04	41.34	271.38	236.73	508.11

Total water input was calculated as the sum of rainfall and applied irrigation during the irrigation-managed period. Progressive (PI), deficit (DI), and extreme deficit irrigation (EDI) were imposed as fixed fractions of the Full treatment (75%, 50%, and 25%, respectively).

Weather data and ETo: Daily reference evapotranspiration (ETo) was calculated using the FAO-56 Penman–Monteith method (Allen et al., 1998, see **Supplementary Methods S1**) based on meteorological data obtained from the USDA-NRCS SCAN weather station (Site #2223) located at the George Washington Carver Farm. The carbaryl-based insecticide Sevin was applied annually to control *Lygus lineolaris.*

### 2.5 Data collection

Meteorological data (air temperature, rainfall, and relative humidity) were recorded at the nearest USDA–NRCS weather station (Site #2223), and the date of heavy rain was noted in both years. Plant height, biomass, disease score, and grain yield were measured as primary agronomic response variables in both cropping seasons, using 5–10 representative plants per plot. At maturity, three randomly selected plants from the middle row of 72 plots were harvested, threshed, cleaned, weighed, and averaged to calculate yield per plant (g plant^−1^). Disease severity was visually assessed on a 1–5 ordinal scale, with 1 indicating a plant free of visible disease symptoms and 5 indicating severe symptoms resulting in plant death. Disease ratings were recorded on representative plants within each plot during peak disease incidence. Physiological measurements were made on fully expanded, sun-exposed leaves with a LI-6800 portable photosynthesis system (LI-COR Biosciences, Lincoln, NE, USA) under controlled conditions of 400 µmol mol^−1^ CO_2_, 30 °C leaf temperature, 60–65% relative humidity, and a photosynthetic photon flux density (PPFD) of 1000 µmol m^−2^ s^−1^. Measurements were taken on one representative plant per plot on multiple days after planting in both growing seasons. Stomatal conductance was also measured on five plants per plot with a LI-600 porometer under high irradiance (>800 µmol m^−2^ s^−1^). Leaf chlorophyll content was estimated with a SPAD-502Plus meter (Konica Minolta, Tokyo, Japan) on two leaves per plant (upper and lower canopy) at key growth stages, and crop phenology (bud initiation, flowering, and maturity) was recorded throughout the season. Agronomic efficiency of nitrogen (AE_n_) was calculated as the increase in grain yield relative to the zero-nitrogen control divided by the amount of nitrogen applied (AE_n_ = (Y_n_ − Y_0_)/N), following established nitrogen-use efficiency frameworks (Fixen et al., 2015; Omara et al., 2019; Congreves et al., 2021).

where Y_n_ is the grain yield at a given nitrogen application rate, Y0 is the grain yield of the unfertilized control (0 kg N ha^−1^), and N is the amount of N applied in kg N ha^−1^. UAS acquisition and processing: The Matrice 350 RTK, equipped with a MicaSense RedEdge-MX Dual multispectral system, was used to collect UAS data. Multispectral imagery was radiometrically calibrated using sensor-specific calibration panels collected before and after each flight, then processed into georeferenced orthomosaics and surface reflectance products. Flights were conducted under clear-sky conditions near solar noon at 20–25 m above ground level, with 80% forward and 70% side overlap. Real-time kinematic (RTK) positioning was enabled, and four fixed ground targets were deployed to support accurate georeferencing. Multiple flights per season were conducted from early vegetative through preharvest stages. NDVI was calculated from reflectance mosaics, aggregated at the plot level using mean pixel values within plot boundaries, and used to assess canopy status and stress alongside field physiological measurements. For this study, NDVI analyses were restricted to the 2025 growing season because UAS flight timing and temporal coverage were most consistent and complete in 2025, enabling a robust, stage-resolved assessment of canopy dynamics. NDVI extraction employed an inward-buffered, interior-pixel method to minimize edge effects and spectral contamination, and plot-level values were calculated as the mean of the retained interior pixels.

### 2.6 Statistical analysis

Morphological traits (plant height, NDVI, and grain yield) and physiological traits (net photosynthetic rate, stomatal conductance, and related gas-exchange parameters) were analyzed using mixed-effects analysis of variance (ANOVA). NDVI measurements from the 2025 growing season were included in this analysis. This restriction ensured uniform temporal sampling across days after planting and preserved the integrity of NDVI-derived canopy dynamics by avoiding variability in flight timing. Genotype, irrigation regime, and nitrogen rate were specified as fixed effects, while year and replication were treated as random effects. Variation in nitrogen application timing across years was accounted for by including year as a random factor. Model assumptions of normality and homogeneity of variances were assessed using the Shapiro–Wilk, Brown–Forsythe, and Levene tests. Statistical analyses were performed using JMP Pro 18 (SAS Institute Inc., Cary, NC, USA). Variables that deviated from normality, including vapor pressure deficit (VPD), were log-transformed prior to analysis. Data visualization and figure generation were performed using Python. When significant main effects or interactions were identified, mean separation was performed using Tukey’s honestly significant difference (HSD) test at α = 0.05.

## 3 Results

### 3.1 Seasonal water inputs across irrigation regimes

Seasonal water inputs (rainfall + irrigation) were quantified to verify treatment separation under humid Midwest conditions (**Table 3**). In 2024, total seasonal rainfall during the irrigation-managed period was 338.33 mm. Total irrigation applied ranged from 353.58 mm under Full irrigation to 222.38 mm under extreme deficit irrigation (EDI), resulting in corresponding total water inputs of 691.91 mm (Full), 648.04 mm (PI), 604.58 mm (DI), and 560.71 mm (EDI). In 2025, total seasonal rainfall amounted to 236.73 mm, while irrigation ranged from 396.18 mm under Full irrigation to 271.38 mm under EDI. This produced total water inputs of 632.91 mm (Full), 591.18 mm (PI), 549.84 mm (DI), and 508.11 mm (EDI). Despite substantial rainfall contributions in both seasons, clear and consistent separation in total water input was maintained among irrigation treatments (Full > PI > DI > EDI), confirming that irrigation gradients were successfully imposed during the late-season treatment window under humid temperate conditions.

### 3.2 Physiological responses of quinoa to irrigation and nitrogen

#### 3.2.1 Effects of irrigation, fertilizer, and variety on gas-exchange traits

Analysis of variance (ANOVA) demonstrated that gas-exchange responses varied across growth stages and were differentially regulated by irrigation, nitrogen (N), and genotype (**Table 1**). At bud initiation, irrigation significantly affected stomatal conductance (gsw), transpiration (E), net photosynthetic rate (A), and intercellular CO_2_ concentration (Ci) (P < 0.05), whereas N significantly influenced only Ci. Genotypes significantly affected E, A, and Ci, and a significant genotype × irrigation interaction was detected for gsw and Ci (P < 0.05). Interaction means (**Supplementary Table 5**) showed that Var1 exhibited the highest gsw under Full irrigation (2.02 mol m^−2^ s^−1^) but declined sharply under deficit irrigation (0.66 mol m^−2^ s^−1^), indicating strong stomatal sensitivity to reduced water supply. In contrast, Var2 maintained more stable gsw across irrigation regimes, with peak conductance under progressive irrigation (1.68 mol m^−2^ s^−1^). Similar genotype-dependent patterns were observed for Ci, with Var1 showing reduced Ci under deficit irrigation relative to Full irrigation, whereas Var2 maintained relatively consistent Ci values. These results indicate tighter stomatal regulation and greater hydraulic sensitivity in Var1 during early vegetative development.

During flowering, irrigation significantly affected gsw and E, while N influenced A (P < 0.05), and no significant interaction effects were detected (**Table 1**). Although statistical interactions were absent at this stage, irrigation-driven differences in stomatal conductance remained physiologically evident under deficit treatments.

At grain filling, nitrogen became the dominant regulatory factor, significantly affecting gsw, E, and A (P < 0.01), with strong irrigation × N interactions (P < 0.01), while irrigation alone significantly influenced A (**Table 1**). Genotype significantly affected gsw and E, and a significant genotype × irrigation interaction was detected for Ci (P < 0.01). Means separation revealed clear divergence under severe water limitation (**Supplementary Table 5**). The highest photosynthetic rates were observed under DI × 150 kg N ha^−1^ (33.83 µmol m^−2^ s^−1^) and EDI × 150 kg N ha^−1^ (34.75 µmol m^−2^ s^−1^), whereas the lowest A occurred under DI × 0 kg N ha^−1^ (17.08 µmol m^−2^ s^−1^). Stomatal conductance and transpiration followed similar trends, peaking under DI × 150 kg N ha^−1^ (**Supplementary Table 6**). In contrast, Var1 exhibited markedly lower Ci under extreme deficit irrigation (137.48 ppm), whereas Var2 maintained higher Ci values under deficit treatments (195–205 ppm), indicating contrasting physiological adjustments during reproductive development. Collectively, these results indicate that irrigation effects were most pronounced during early vegetative growth, whereas nitrogen regulation and irrigation × N interactions dominated gas-exchange dynamics during grain filling, reflecting a developmental shift in the primary controls of photosynthetic performance.

Least-squares means showed no significant effect of nitrogen application on physiological traits at bud initiation, indicating that early vegetative gas exchange (A, gsw, Ci, and E) was not strongly regulated by nitrogen supply (**Table 4**). However, during the grain-filling stage, least-squares means for photosynthetic rate, stomatal conductance, and transpiration rate increased significantly under 75 and 150 kg N ha^−1^. At the same time, intercellular CO_2_ concentration decreased relative to 0 N, reflecting enhanced stomatal regulation and carbon assimilation with increasing nitrogen availability (**Table 4**). These physiological responses were associated with greater least-squares mean biomass accumulation and grain yield at maturity, with the highest values observed at 150 kg N ha^−1^. In contrast, nitrogen-deficient plants exhibited reduced growth and productivity (**Table 4**). Disease severity increased significantly with nitrogen rate (**Table 4**). Plants receiving 150 kg N ha^−1^ exhibited the highest disease scores, followed by 75 kg N ha^−1^, whereas unfertilized plants showed the lowest disease severity. These results indicate that higher nitrogen availability was associated with increased disease expression, coinciding with greater biomass accumulation and canopy development at higher nitrogen rates.

**Table 4:** Least-squares means of leaf physiological traits, including net photosynthetic rate (A), stomatal conductance to water vapor (gsw), intercellular CO_2_ concentration (Ci), and transpiration rate (E), measured at key quinoa growth stages, and morphological traits (aboveground biomass, disease score, and grain yield per plant (g plant^−1^)) at harvest, as affected by nitrogen fertilizer rate.

Nitro. rate (kg ha^−1^)	Bud initiation				Grain filling				Maturity	Morphology		
	A	gsw	Ci	E	A	gsw	Ci	E	A	Biomass	Disease score	Yield (g/plant)
0	30.4a	1.5a	290.2a	11.9a	20.1b	0.23b	197.7a	8.8b	24.9b	237.8b	3.51c	39.2b
75	30.3a	1.3ab	279.7a	11.8a	26.4a	0.35a	179.3a	11.6a	25.3b	336.9a	3.8b	59.9a
150	28.4a	1.0a	276.5a	10.8a	28.8a	0.36a	171.8a	11.4a	27.8a	354.4a	4.0a	68.1a

Different lowercase letters within a column indicate significant differences at P ≤ 0.05 (Tukey’s HSD).

**Figure 3:**
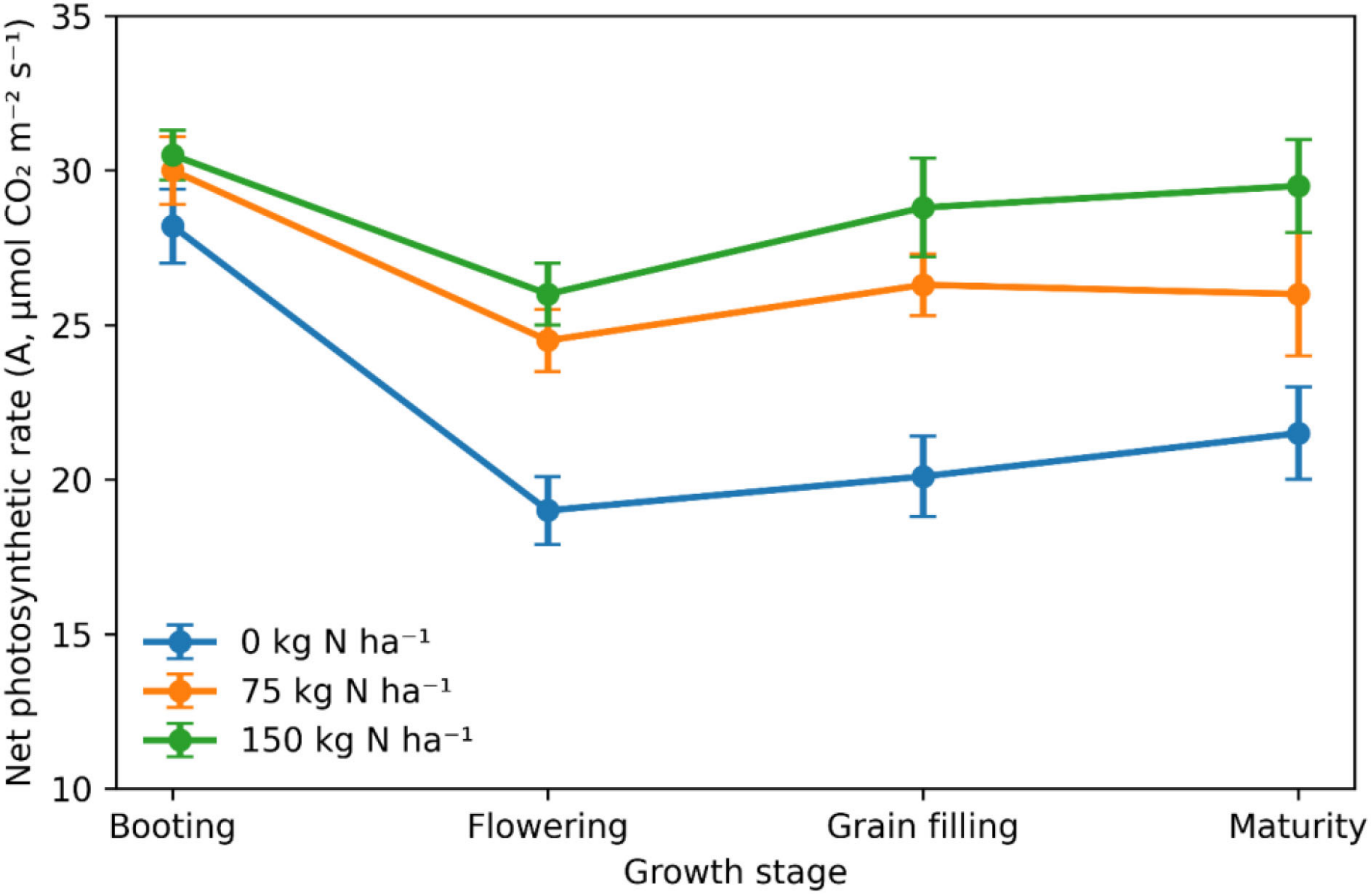
Net photosynthetic rate (A) of quinoa across growth stages as influenced by nitrogen application rates (0, 75, and 150 kg N ha^−1^). Values represent means ± SE. The increase in photosynthetic rate during grain filling reflects improved late-season microclimatic conditions (lower temperature and vapor pressure deficit) and sustained nitrogen support of leaf-level photosynthetic capacity, rather than a stay-green effect or delayed senescence.

Photosynthetic rate varied across growth stages and nitrogen levels. Photosynthesis rose from 28.35 µmol m^−2^ s^−1^ with no nitrogen to 30.47 and 30.39 µmol m^−2^ s^−1^ at 75 and 150 kg N ha^−1^, respectively. A decline occurred during flowering, especially under no nitrogen, when photosynthesis fell to 18.98 µmol m^−2^ s^−1^. During grain filling and maturity, photosynthesis recovered, reaching 26.36–28.83 µmol m^−2^ s^−1^ and 26.10–29.49 µmol m^−2^ s^−1^ under 75–150 kg N ha^−1^. The stage-dependent increase in photosynthetic capacity with increasing nitrogen supply reflects enhanced nitrogen utilization, thereby supporting carbon assimilation (**Figure 3**).

**Figure 4:**
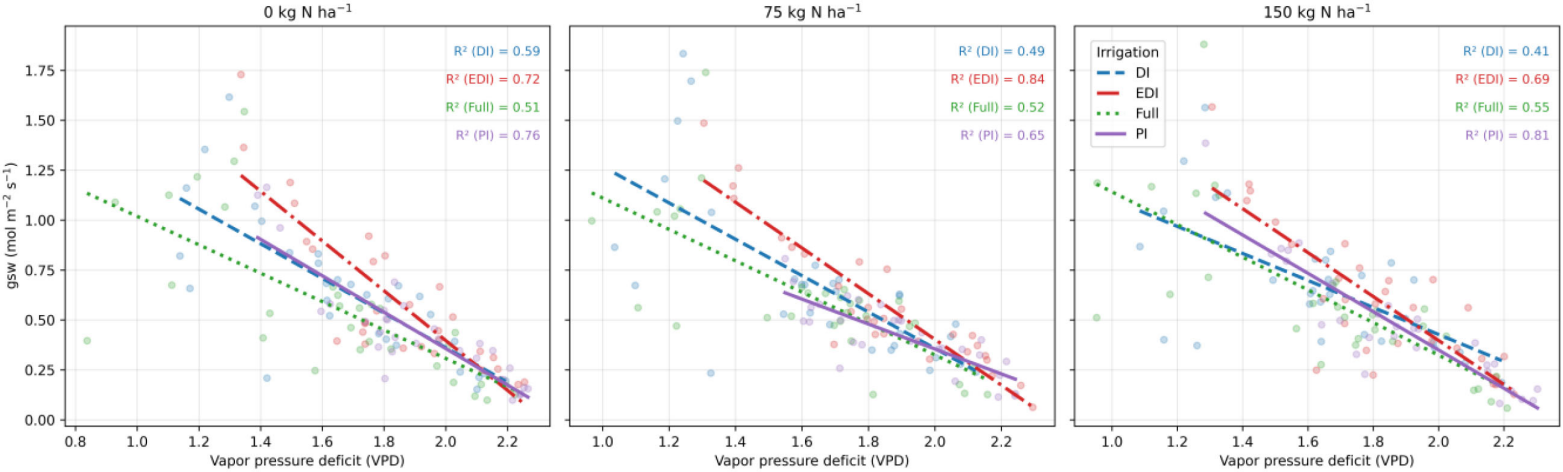
Response of stomatal conductance (gsw) to vapor pressure deficit (VPD) under different irrigation regimes and nitrogen (N) rates in quinoa grown under humid temperate conditions. Panels show N application rates of 0, 75, and 150 kg N ha^−1^. Points represent individual observations and lines indicate irrigation-specific linear regressions with R^2^ values reported within each panel.

Pre-plant soil analyses indicated a slightly acidic to near-neutral pH (5.6–6.4), low organic matter (0.8–1.2%), and moderate to high CEC (9.6–18.6 meq 100 g^−1^). Initial inorganic nitrogen concentrations were uniformly low across plots, with NO_3_^−^–N ranging from 1.38 to 2.02 ppm and NH_4_^+^–N from 3.86 to 9.67 ppm (**Supplementary Table 4**). Across nitrogen levels (0, 75, and 150 kg N ha^−1^), stomatal conductance (gsw) declined linearly with increasing vapor pressure deficit (VPD) under all irrigation regimes (**Figure 4**), indicating strong atmospheric control of stomatal behavior. The strength of the gsw–VPD relationship varied across irrigation treatments and nitrogen rates. At 0 kg N ha^−1^, the relationship was strongest under progressive irrigation (PI; R^2^ = 0.76) and extreme deficit irrigation (EDI; R^2^ = 0.72), followed by deficit irrigation (DI; R^2^ = 0.59), with full irrigation showing a weaker association (R^2^ = 0.51). At 75 kg N ha^−1^, the strongest coupling between gsw and VPD occurred under EDI (R^2^ = 0.84), with moderate relationships under PI (R^2^ = 0.65), DI (R^2^ = 0.49), and full (R^2^ = 0.52). Similarly, at 150 kg N ha^−1^, PI (R^2^ = 0.81) and EDI (R^2^ = 0.69) exhibited strong gsw–VPD relationships, whereas DI and full irrigation showed lower explanatory power (R^2^ = 0.41 and 0.55, respectively). Overall, stomatal sensitivity to atmospheric demand was consistently pronounced under deficit-oriented irrigation regimes (PI and EDI) and weakest under full irrigation, highlighting the dominant role of VPD in regulating gsw under combined soil and atmospheric water stress.

**Figure 5:**
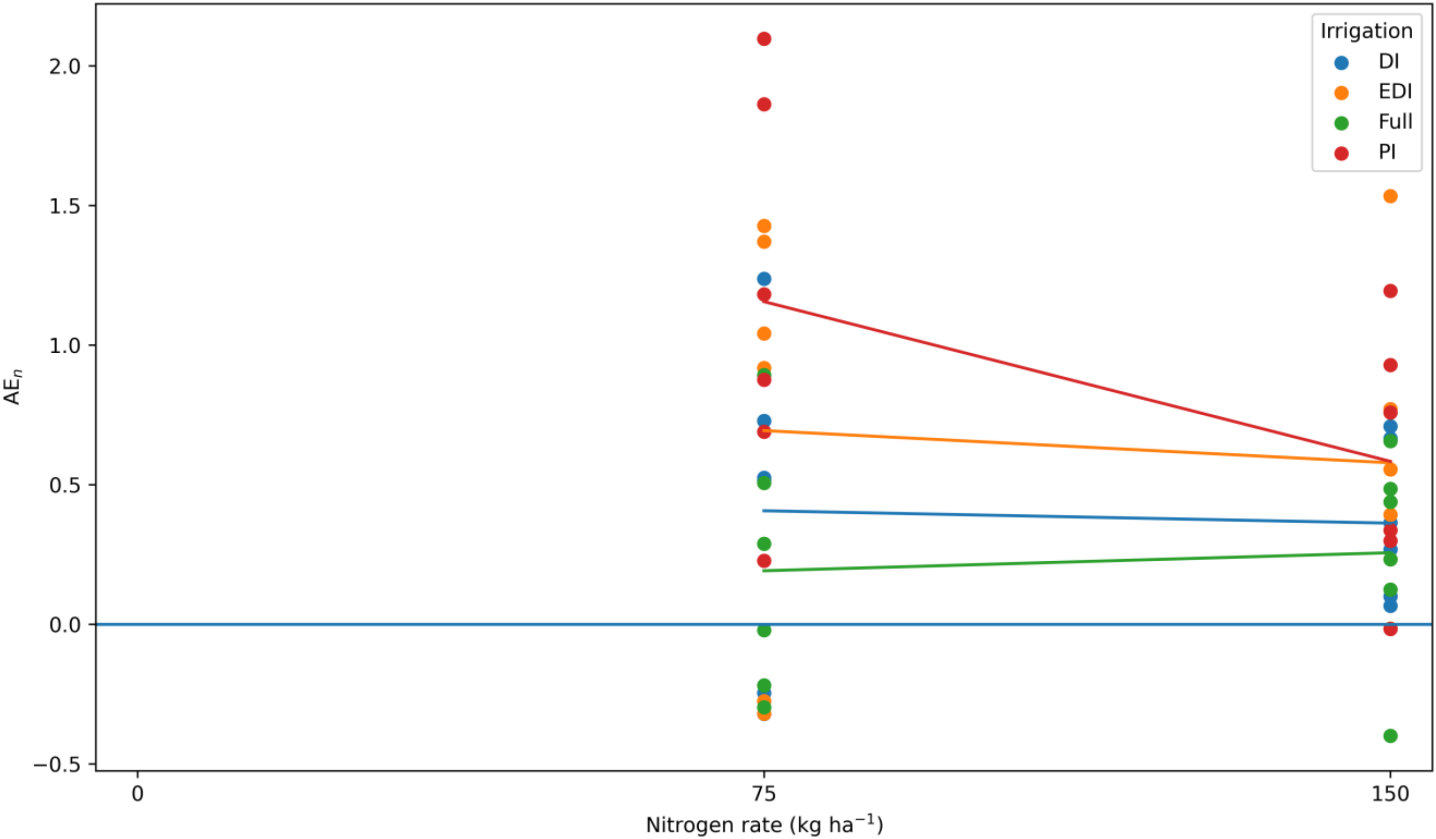
Agronomic efficiency of nitrogen (AE_n_) of quinoa is influenced by irrigation regime and nitrogen rate. Agronomic efficiency of nitrogen (AE_n_) under full irrigation (Full), progressive irrigation (PI), deficit irrigation (DI), and extreme deficit irrigation (EDI) at 75 and 150 kg N ha^−1^. Bars indicate treatment means and show the interaction between nitrogen rate and irrigation regime.

Agronomic efficiency of nitrogen (AE_n_) varied among irrigation regimes, whereas nitrogen rate did not show a significant main effect (**Figure 5**; **Supplementary Table 3**). Across nitrogen rates, progressive irrigation (PI) generally had higher AE_n_ than extreme deficit irrigation (EDI) and deficit irrigation (DI), while full irrigation consistently produced lower AE_n_. Although AE_n_ under PI tended to decline as nitrogen rate increased from 75 to 150 kg N ha^−1^, changes in AE_n_ across nitrogen rates within individual irrigation treatments were relatively small. The absence of a significant nitrogen × irrigation interaction indicates that observed differences in AE_n_ were primarily driven by irrigation regime rather than differential responses to nitrogen rate.

#### 3.2.2 NDVI responses to nitrogen application across growth stages

**Figure 6:**
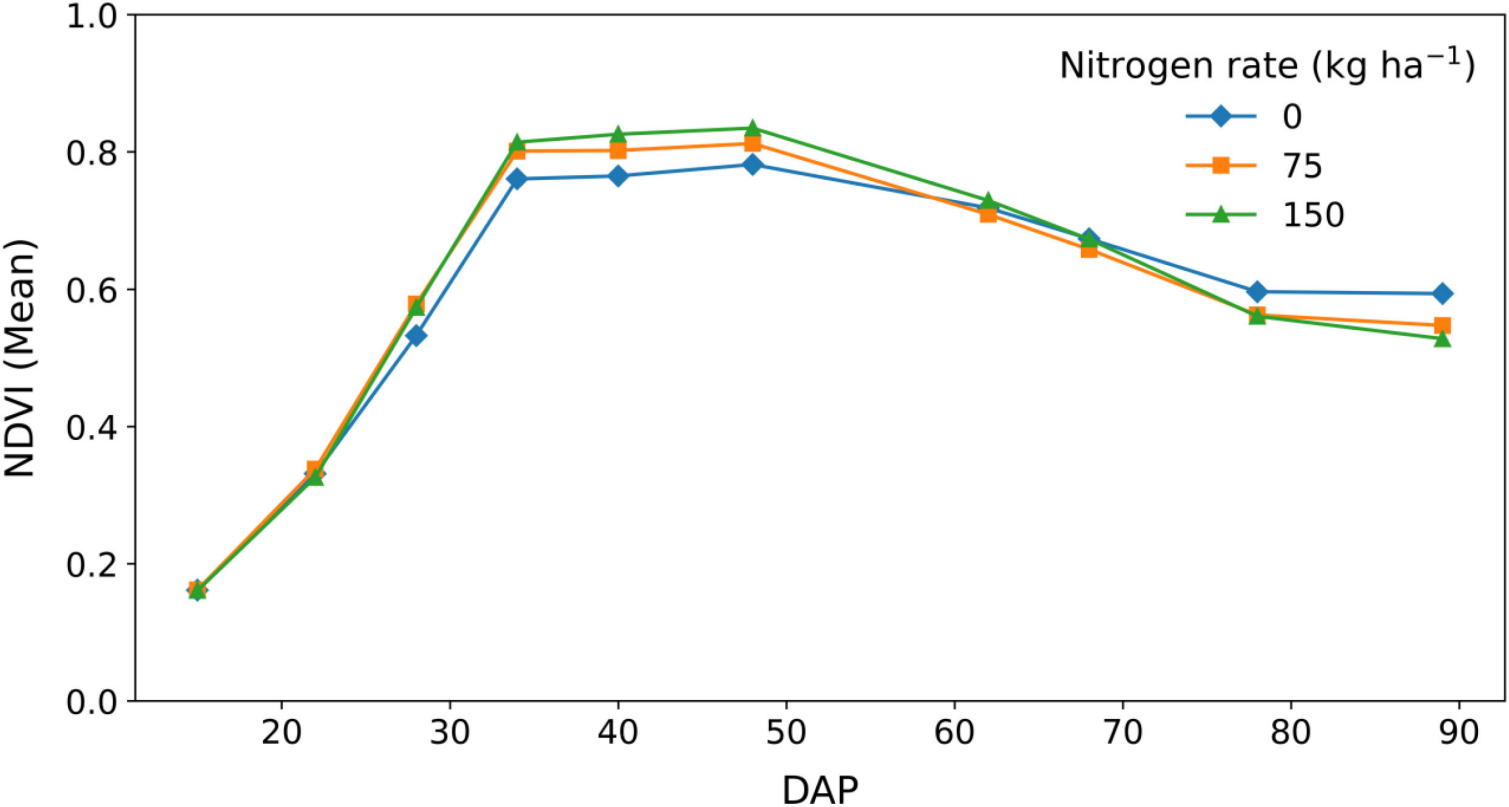
Temporal changes in the normalized difference vegetation index (NDVI) of quinoa measured on different days after planting (DAP) under three nitrogen application rates (0, 75, and 150 kg N ha^−1^). NDVI increased with crop development, reached peak values during mid-season canopy growth (34–48 DAP), and declined toward later growth stages. Higher nitrogen rates resulted in greater NDVI during peak growth, while treatment differences were minimal during early establishment and late-season senescence. Values represent means ± (SE).

Normalized difference vegetation index (NDVI) varied with crop developmental stage and nitrogen application rate (**Figure 6**). Across all treatments, NDVI was low during early growth stages (15–22 DAP), ranging from approximately 0.15 to 0.35, and increased rapidly between 22 and 34 DAP, coinciding with active vegetative canopy development. Peak NDVI occurred during mid-season growth (34–48 DAP), ranging from approximately 0.75 under 0 kg N ha^−1^ to 0.82–0.85 under 75 and 150 kg N ha^−1^. During this period, higher nitrogen rates consistently produced greater NDVI, with 150 kg N ha^−1^ showing the highest canopy greenness, followed by 75 kg N ha^−1^, while unfertilized plots maintained lower NDVI. The statistical results in **Supplementary Table 1** support these nitrogen-related differences during peak canopy development. After 48 DAP, NDVI declined steadily across all nitrogen treatments, reflecting progressive canopy senescence. By late growth stages (78–89 DAP), NDVI values converged across treatments (approximately 0.52–0.60), indicating reduced nitrogen effects as crop maturity approached.

### 3.2 Morphological responses of quinoa to irrigation and nitrogen

#### 3.2.1 Plant height

Plant height responses varied between years, reflecting differences in phenological coverage and sampling intensity (**Table 5**). In 2024, irrigation significantly affected plant height at the vegetative (P < 0.001), flowering (P < 0.001), and maturity stages (P < 0.0001). Variety effects were detected only at maturity (P < 0.01), while nitrogen showed no significant main effects at any growth stage. Significant irrigation × variety interactions occurred at flowering (P < 0.05) and maturity (P < 0.0011), indicating genotype-specific responses to water availability, whereas irrigation × nitrogen, nitrogen × variety, and three-way interactions were not significant. In 2025, irrigation significantly influenced plant height at bud initiation (P < 0.001), vegetative growth (P < 0.0001), and flowering (P < 0.01), but not at maturity. Nitrogen exerted significant effects across all measured stages (P < 0.05), and variety effects were pronounced at flowering and maturity (P < 0.0001). Significant irrigation × variety interactions at the vegetative (P < 0.001) and flowering stages (P < 0.05), as well as nitrogen × variety interactions at bud initiation (P < 0.05), vegetative (P < 0.05), and flowering (P < 0.01), further demonstrate genotype-specific sensitivity to water and nitrogen supply. No significant irrigation × nitrogen or three-way interactions were detected, suggesting largely additive effects of water and nitrogen across genotypes.

**Table 5:** Analysis of variance (ANOVA) results for plant height of quinoa across growth stages under irrigation (Irr), nitrogen fertilizer (Fert), and variety (Var) treatments during the growing seasons. Values represent *P*-values for main effects and interactions.

Source	2024			2025				
	Veg	Flow	Mat	Brn	Budi	Veg	Flow	Mat
Var	0.151	0.3507	0.05	0.5259	0.0749	0.1057	0.001	0.001
Irr	0.01	0.05	0.001	0.8208	0.001	0.001	0.01	0.149
Nitro.	0.3031	0.9091	0.7903	0.5895	0.001	0.001	0.05	0.001
Var x Irr	0.4892	0.05	0.01	0.9091	0.0569	0.001	0.05	0.5764
Irr x Nitro.	0.9616	0.9018	0.8445	0.9964	0.9954	0.9268	0.2206	0.7813
Var x Nitro.	0.6752	0.8141	0.5045	0.9788	0.05	0.05	0.05	0.8837
Var x Irr x Nitro.	0.8858	0.7599	0.576	1.00	0.8857	0.5295	0.3292	0.9607

Significance levels are indicated as *P* < 0.05, *P* < 0.01, and *P* < 0.001. In 2024, plant height data were collected at vegetative (Veg), flowering (Flow), and maturity (Mat) stages, whereas additional growth stages, such as branching (Brn) and bud initiation (Budi), were assessed in 2025.

**Figure 7:**
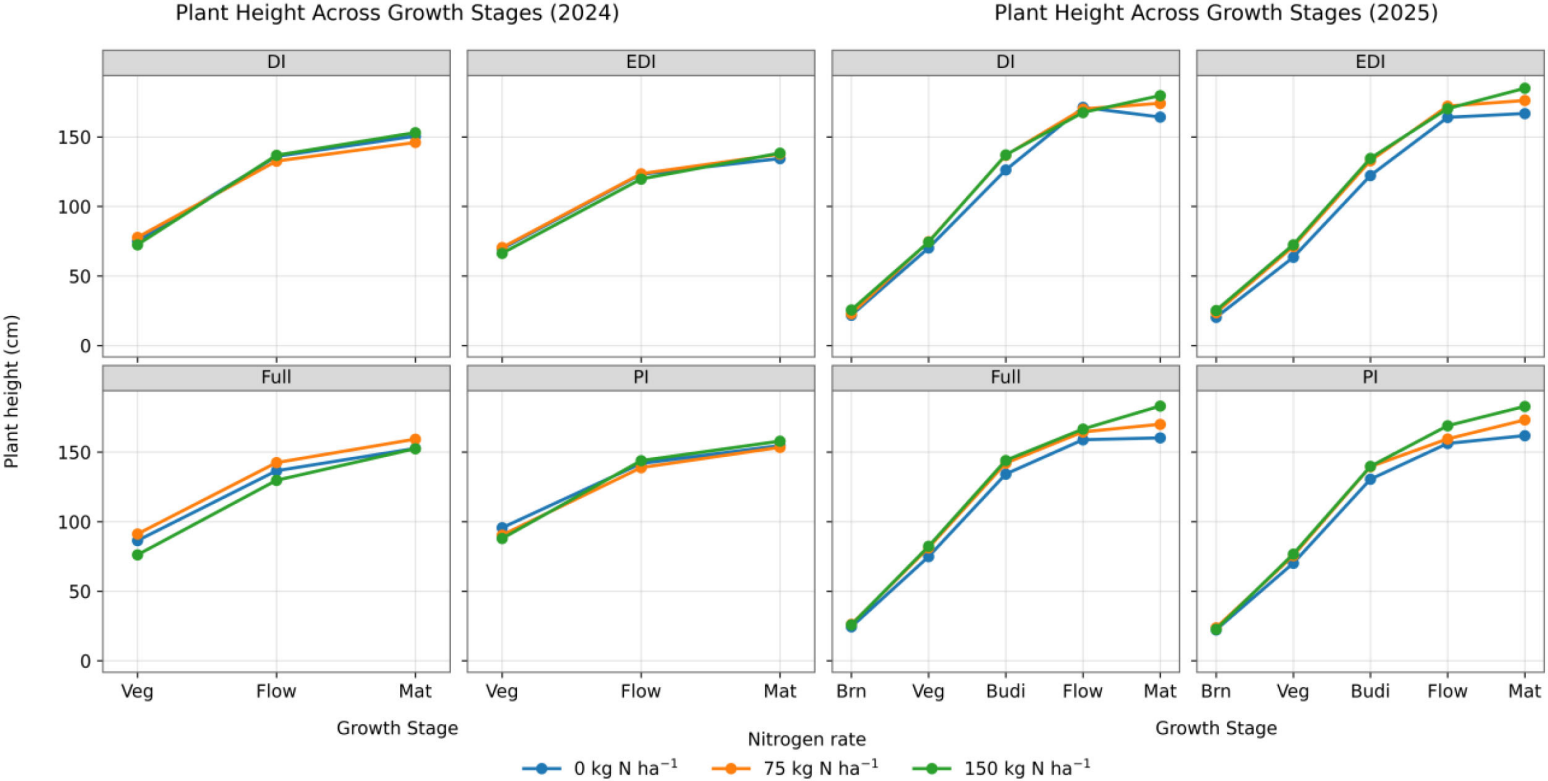
Growth-stage dependent responses of quinoa plant height to nitrogen supply across irrigation regimes in 2024 and 2025. Lines represent mean plant height for 0, 75, and 150 kg N ha^−1^. Veg, Vegetative; Flow, Flowering; Brn, Branching; Budi, Bud initiation; Mat, Maturity.

Plant height increased progressively with advancing growth stage in both years across all irrigation regimes (**Figure 7**). In 2024, quinoa plants showed rapid height gain from the vegetative to flowering stage, followed by smaller increases toward maturity. Nitrogen application consistently promoted taller plants, though differences among nitrogen rates diminished by maturity. In 2025, finer phenological sampling revealed a sharp increase in plant height from branching to budding, followed by a gradual plateau from flowering to maturity. Higher nitrogen rates increased plant height throughout development, with the most significant separation among nitrogen treatments during budding and flowering. Differences between irrigation regimes remained modest, and trajectories were essentially parallel. These visual trends are supported by the ANOVA results (**Table 5**), which indicate dominant effects of growth stage and nitrogen, with comparatively weaker effects of irrigation on plant height.

#### 3.2.2 Grain yield

The factorial ANOVA revealed that nitrogen availability was the dominant factor influencing quinoa grain yield, with a highly significant main effect (P < 0.001; **Supplementary Table 2**). Grain yield differed significantly among genotypes across treatments (P < 0.05). The high-yielding genotype consistently produced higher grain yield across irrigation and nitrogen treatments, whereas the low-yielding genotype consistently produced lower grain yield. The main effect of irrigation was not statistically significant, suggesting that the irrigation regime alone did not uniformly alter grain yield under the conditions of this study. None of the two-way interactions (irrigation × nitrogen, irrigation × variety, or nitrogen × variety) was significant (P < 0.05), indicating that the effects of nitrogen and variety were largely consistent across irrigation regimes. Similarly, the three-way interaction among irrigation, nitrogen, and variety was not significant, demonstrating that varietal yield responses to nitrogen did not depend on irrigation treatment (**Supplementary Table 1**).

**Figure 8:**
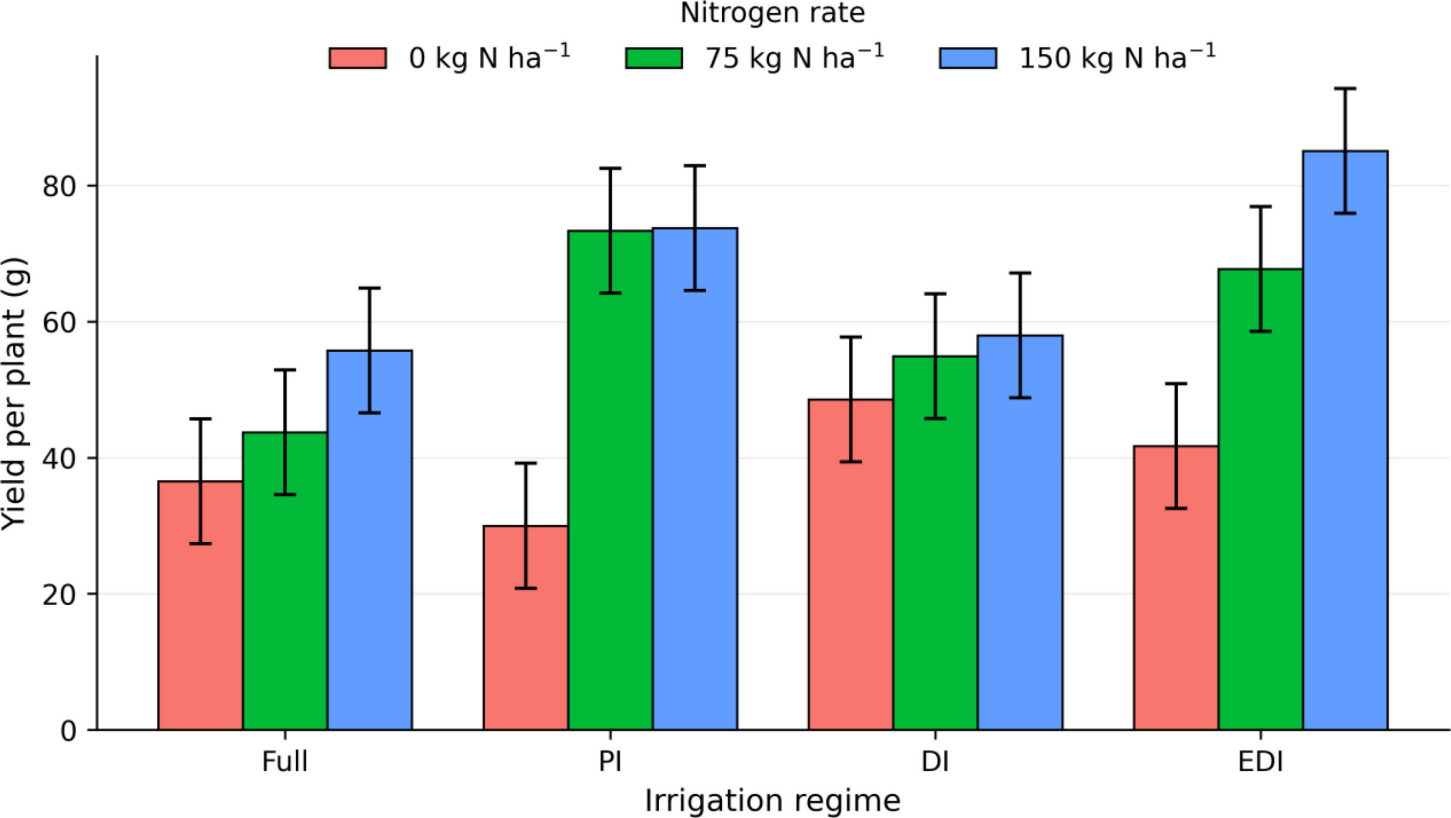
Interaction effects of irrigation regime and nitrogen rate on quinoa grain yield. Bars represent means averaged across the 2024 and 2025 growing seasons; error bars indicate pooled standard error derived from the residual mean square of the ANOVA. Full irrigation (Full), partial irrigation (PI), deficit irrigation (DI), and extreme deficit irrigation (EDI), fertilizer rate 0, 75, and 150 kg N ha^−1^.

Grain yield increased significantly with nitrogen fertilization across all irrigation regimes (**Figure 8**). Under full irrigation, both 75 and 150 kg N ha^−1^ exceeded the control (zero nitrogen application), though the difference between the two nitrogen levels was small. In contrast, nitrogen-driven yield increases were more pronounced under progressive, deficit, and extreme deficit irrigation, with the highest yield observed under extreme deficit irrigation with 150 kg N ha^−1^. Despite these findings, the irrigation × nitrogen interaction was not statistically significant, indicating that nitrogen effects remained consistent in direction across irrigation regimes, with irrigation affecting the magnitude but not the overall pattern of nitrogen responses.

## 4 Discussion

The results of this study demonstrate developmental reprogramming of gas-exchange regulation in quinoa, with the relative importance of water and nitrogen constraints shifting across growth stages. Irrigation exerted dominant control over physiological performance during early development and flowering, whereas nitrogen availability and its interaction with water emerged as the primary regulators during grain filling. Quinoa exhibited clear physiological sensitivity to water availability during flowering, as evidenced by reductions in stomatal conductance and leaf-level gas exchange under deficit irrigation, indicating that flowering and fruit set are water-sensitive phases. Similar sensitivity has been reported previously in quinoa and reflects well-established hydraulic and atmospheric constraints on carbon assimilation in C_3_ crops (Razzaghi et al., 2012; Hinojosa et al., 2018; Chaves et al., 2009; Li et al., 2022). Under the humid-temperate conditions of Missouri, water availability during grain filling exerted a stronger influence on yield stability and nitrogen agronomic efficiency than during earlier growth stages, indicating that post-anthesis development is the primary physiological control point.

Under humid-temperate field conditions, rainfall can substantially contribute to crop water supply and may partially offset reductions in supplemental irrigation; however, its distribution is often uneven and may not coincide with physiologically sensitive stages (Hinojosa et al., 2018; Sinclair and Rufty, 2012). In the present study, precipitation was non-uniform, with relatively modest rainfall during the late-season irrigation treatment window, particularly in 2025. Seasonal water budgets were therefore quantified to verify treatment differentiation (**Table 3**). Although rainfall contributed 338.33 mm in 2024 and 236.73 mm in 2025, clear separation in realized total water input was maintained among irrigation treatments (Full > PI > DI > EDI), confirming that irrigation gradients were effectively imposed despite the humid environment.

Imposing differential irrigation during early vegetative growth in humid temperate systems is often constrained by unpredictable rainfall, thereby limiting precise control of soil moisture. In contrast, the late-season period from late July through September is typically drier and coincides with quinoa’s reproductive and grain-filling stages, periods during which water availability strongly regulates carbon assimilation, canopy function, and nitrogen utilization (Hinojosa et al., 2019; Razzaghi et al., 2012). Thus, the timing of irrigation treatment initiation reflects both agro-climatic feasibility and biological relevance rather than experimental limitations.

Although cumulative seasonal water inputs occasionally exceeded the ET_0_-based supplemental irrigation target of 430 mm, this value represents a planned irrigation allocation rather than total crop water demand. Rainfall effectiveness is constrained by its temporal distribution, soil water storage capacity, and atmospheric evaporative demand (Sinclair and Rufty, 2012). Irrigation, therefore, functioned primarily to stabilize water availability during dry intervals and periods of elevated vapor pressure deficit. The absence of a strong irrigation main effect on grain yield thus reflects quinoa’s resilience to moderate late-season reductions in supplemental irrigation rather than failure to impose water gradients. This interpretation is supported by treatment separation in realized water inputs (**Table 3**) and by concurrent physiological adjustments in stomatal conductance, canopy vigor, and nitrogen-use efficiency observed in this study, consistent with established mechanisms linking post-anthesis water and nitrogen availability to carbon assimilation and yield formation in C_3_ crops (Hinojosa et al., 2019; Sinclair and Rufty, 2012).

During bud initiation, irrigation significantly affected all gas-exchange parameters in this study, indicating that early quinoa growth was strongly constrained by plant water status. This sensitivity aligns with the tight coupling among leaf water potential, stomatal conductance, and internal CO_2_ diffusion before full canopy development (Flexas et al., 2004; Chaves et al., 2009). Pronounced irrigation effects on intercellular CO_2_ concentration (Ci) and net photosynthesis (A) further suggest that both diffusive and biochemical limitations restrict carbon uptake under early-season water stress. Varietal effects were observed for transpiration, photosynthesis, and intercellular CO_2_ concentration, but not for stomatal conductance, indicating that genotypic differences at this stage were primarily expressed at the biochemical level rather than through stomatal regulation. Such functional decoupling between stomatal conductance and photosynthesis has been reported in quinoa and other stress-tolerant species, where variation in stomatal conductance and photosynthetic capacity drives differences in intrinsic water-use efficiency (Razzaghi et al., 2012). The significant interaction between variety and irrigation further indicates genotype-specific sensitivity to soil moisture during early development, which may contribute to differential early-season drought adaptation.

Nitrogen effects during bud initiation were limited to changes in intercellular CO_2_ concentration, without corresponding increases in net photosynthesis. This pattern is characteristic of early developmental stages that are sink-limited, in which increased nitrogen availability does not immediately translate into greater photosynthetic capacity or Rubisco investment (Evans, 1989; Sinclair and Rufty, 2012).

In contrast, grain filling emerged as the most physiologically sensitive stage under combined water and nitrogen constraints in this study. During this phase, nitrogen significantly increased stomatal conductance, transpiration, and net photosynthesis. The strong interaction between irrigation and nitrogen indicates that both resources together limit carbon assimilation. Nitrogen-driven increases in photosynthetic rate likely reflect enhanced Rubisco content and electron transport capacity, consistent with well-established responses in C_3_ crops (Evans, 1989; Li et al., 2022; Deng et al., 2024). Consistent with these responses, net photosynthetic rate during grain filling was higher than during flowering in this study, despite concurrent declines in canopy greenness and leaf area, indicating that this response does not represent a stay-green phenotype or delayed senescence. Field observations from this study indicate that grain filling coincided with more favorable late-season microclimatic conditions in Missouri, characterized by lower temperatures and reduced vapor pressure deficit, which likely alleviated stomatal limitation and enhanced instantaneous carbon assimilation. Adequate nitrogen availability during this stage further supported leaf-level photosynthetic capacity, as evidenced by higher photosynthetic rates under fertilized treatments despite advancing canopy senescence. Importantly, the convergence of NDVI values among nitrogen treatments at later growth stages observed in this study confirms that increased nitrogen supply did not extend canopy persistence but rather enhanced peak and late-season photosynthetic performance of remaining functional leaves. These patterns are consistent with established physiological mechanisms linking water status, nitrogen availability, and post-anthesis carbon assimilation in quinoa and other C_3_ crops (Chaves et al., 2009; Razzaghi et al., 2012; Grossiord et al., 2020; Hinojosa et al., 2019).

The absence of strong genotype-specific interactions at this stage further suggests that management practices exert a greater influence on grain filling than inherent genetic differences. This indicates that grain filling represents a critical window for agronomic refinement, in which strategic adjustments in irrigation and nitrogen management may be more effective than genotype-specific interventions under the conditions evaluated.

During flowering, irrigation again emerged as the dominant control of stomatal conductance and transpiration in this study, indicating tight stomatal regulation that balances carbon gain with hydraulic safety during a critical reproductive phase. This pattern aligns with established stomatal responses that primarily depend on plant water status and atmospheric demand during periods of high hydraulic risk (Chaves et al., 2009; Li et al., 2022). In contrast, nitrogen effects during flowering were confined mainly to net photosynthesis, suggesting an enhancement of biochemical photosynthetic capacity without substantial modification of stomatal behavior. This functional decoupling supports the concept that under water-limited conditions, stomatal regulation constrains leaf-level gas exchange, whereas nitrogen availability primarily modulates enzymatic and metabolic capacity rather than stomatal control (Hinojosa et al., 2018). Across developmental stages, the absence of significant three-way interactions among irrigation, nitrogen, and variety indicates that physiological responses were largely additive rather than synergistic. Irrigation exerted the strongest influence during early development and flowering, while nitrogen effects became most pronounced during grain filling, reflecting a shift from hydraulically driven constraints to nutrient-mediated limitations on carbon assimilation and yield formation.

Stomatal responses to atmospheric demand observed in this study clarified the dominant controls on leaf-level gas exchange in quinoa. Across nitrogen rates, stomatal conductance (gsw) declined with increasing vapor pressure deficit (VPD), indicating that atmospheric demand strongly constrained leaf water fluxes. This response aligns with established hydraulic and hormonal regulation of stomata reported across crops and ecosystems (Chaves et al., 2009; Flexas et al., 2008; Li et al., 2022) and with broader evidence that rising VPD increasingly governs stomatal behavior at the ecosystem scale (Novick et al., 2016). The stronger coupling between VPD and gsw under deficit and progressive irrigation observed in this study indicates enhanced hydraulic sensitivity, whereby declining leaf water potential rapidly restricts stomatal opening as atmospheric demand increases. This atmospheric control, evident under water-limited conditions, constrained carbon assimilation through coordinated hydraulic and biochemical limitations on gas exchange (Sperry et al., 2017; Grossiord et al., 2020), consistent with concurrent reductions in photosynthesis (A) and transpiration (E) at high VPD. In contrast, weaker gsw–VPD relationships under full irrigation suggest reduced hydraulic limitation and diminished dependence on feed-forward hydraulic signaling, reflecting a relaxation of atmospheric constraints on stomatal regulation (Flexas et al., 2008; Li et al., 2022).

Nitrogen supply further modulated stomatal regulation, with the strongest VPD–gsw relationships observed at 75 kg N ha^−1^. This response indicates improved coordination among carbon gain, transpiration control, and nitrogen-use efficiency, enabling effective stomatal regulation without the excessive canopy development typically associated with high nitrogen inputs (Sinclair and Rufty, 2012). Such coordination supports enhanced physiological resilience and drought adaptation in quinoa, consistent with prior reports of genotype- and management-dependent water-use strategies in this crop (Hinojosa et al., 2018).

Low pre-plant inorganic nitrogen levels indicate minimal residual mineral N at planting, providing essential context for interpreting nitrogen treatment effects. However, pre-plant soil analysis also revealed a measurable organic nitrogen pool (**Supplementary Table 4**), suggesting potential for in-season mineralization under humid temperate conditions. Although soil organic matter content was modest (0.8–1.2%), total Kjeldahl nitrogen and organic N concentrations (1036–1177 mg kg^−1^ and 1025–1170 mg kg^−1^, respectively) indicate a meaningful reservoir of mineralizable nitrogen. Seasonal rainfall and moderate temperatures likely enhanced microbial activity and nitrogen mineralization during crop development, thereby increasing plant-available N beyond initial soil-test values. This dynamic nitrogen supply likely contributed to the relatively high baseline productivity of the 0 kg N treatment and helps explain why 75 kg N ha^−1^ was sufficient to approach a yield plateau.

Together with moderate-to-high cation exchange capacity, these baseline soil conditions underscore the interaction between inherent soil nitrogen supply and applied fertilizer inputs in shaping nitrogen-use efficiency and irrigation responses observed in this study. Across irrigation regimes and nitrogen rates, agronomic efficiency of nitrogen (AE_n_) varied substantially, underscoring its strong dependence on water availability. The highest AE_n_ under progressive irrigation at 75 kg N ha^−1^ indicates that moderate water and nitrogen supply maximizes the conversion of applied nitrogen into grain yield under humid temperate field conditions. The decline in AE_n_ at higher nitrogen rates reflects diminishing returns from nitrogen input, consistent with established principles of crop nitrogen economics (Sinclair and Rufty, 2012; Hawkesford, 2014). Yield responses closely paralleled AE_n_ patterns, indicating that nitrogen application beyond 75 kg N ha^−1^ did not produce proportional gains in grain yield. Similar findings in quinoa have shown that excessive nitrogen promotes vegetative growth without corresponding increases in grain yield or nitrogen recovery (Sun et al., 2025). In several irrigation × nitrogen combinations, these diminishing returns progressed to negative AE_n_ values.Negative AE_n_ values occurred primarily under Full irrigation and deficit irrigation (DI and EDI), indicating that both moisture surplus and limitation reduced marginal nitrogen return under humid temperate field conditions. Under Full irrigation, excessive soil moisture likely increased nitrogen losses through leaching and denitrification and may have induced transient root-zone hypoxia, lowering nitrogen recovery efficiency. Although previous studies have documented moisture-induced reductions in nitrogen use in flood-prone systems (Kaur et al., 2020), our results extend this understanding by showing that even under controlled supplemental irrigation in a humid temperate field environment, both over- and undersupply of water can suppress agronomic nitrogen efficiency in quinoa. These findings highlight the strong interaction between soil moisture dynamics and nitrogen response, underscoring that nitrogen management should be aligned with irrigation strategy to maximize resource-use efficiency rather than treated as an independent input. In the present study, increased disease severity at higher nitrogen rates further supports this interpretation, as nitrogen-induced increases in canopy biomass and leaf area likely elevated within-canopy humidity and favored pathogen development, particularly under the humid conditions prevailing in July. The absence of yield differences between 75 and 150 kg N ha^−1^ therefore confirms that higher nitrogen inputs did not translate into additional agronomic benefit. Comparable declines in nitrogen-use efficiency under excessive nitrogen supply have been widely reported across cereal and pseudocereal crops, where surplus nitrogen enhances vegetative growth without proportional increases in reproductive sink strength (Sinclair and Rufty, 2012; Xu et al., 2012).

The high performance of quinoa at 75 kg N ha^−1^ under moderate irrigation, observed in this study, indicates that synchronizing nitrogen availability with soil moisture enhances nitrogen uptake and its partitioning to grain under humid temperate field conditions. This treatment combination was also associated with higher intrinsic water-use efficiency and tighter stomatal regulation, suggesting coordinated control of carbon assimilation and transpiration. Similar physiological linkages among stomatal conductance, photosynthetic capacity, and water-use efficiency have been reported under varying water and nitrogen availability, although not under the exact management regimes evaluated here (Flexas et al., 2008; Razzaghi et al., 2012).

Drone-based NDVI provided an integrative, non-destructive indicator of nitrogen response across developmental stages. The seasonal NDVI trajectory, characterized by rapid early-canopy expansion, a mid-season peak, and a progressive decline during senescence, reflects dynamic changes in canopy structure, green biomass, and chlorophyll content. NDVI is widely recognized as a proxy for leaf area index and photosynthetically active biomass, both of which are strongly influenced by nitrogen availability (Huete et al., 2002; Gamon et al., 1995). Recent UAV-based phenotyping studies in quinoa further demonstrate that NDVI effectively captures spatial and temporal variation in canopy development and drought responses, supporting its use as a robust integrative indicator across environments (Lupa-Condo et al., 2024). The strong sensitivity of NDVI to nitrogen during peak canopy development is consistent with findings from other cropping systems, where chlorophyll- and reflectance-based indices closely track nitrogen-driven changes in canopy vigor and yield formation (Nyandi et al., 2025). However, the convergence of NDVI values among nitrogen treatments during late growth stages indicates that higher nitrogen supply did not substantially delay canopy senescence or confer a pronounced stay-green effect. Instead, nitrogen primarily enhanced peak canopy greenness and photosynthetic capacity during mid-season, rather than extending the duration of green leaf area. As plants transition from vegetative to reproductive development and senescence, NDVI becomes less responsive to nitrogen availability due to leaf aging and nitrogen remobilization to developing sinks (Gitelson et al., 2003). Accordingly, yield differences observed at higher nitrogen rates are more plausibly attributed to enhanced peak photosynthetic performance and canopy vigor during critical growth stages, rather than prolonged canopy persistence late in the season. Collectively, these results highlight NDVI as a reliable indicator of crop nitrogen status during early to mid-season growth, with strong potential to guide in-season nitrogen management and improve nitrogen-use efficiency in quinoa production systems.

Plant height responses indicate that quinoa canopy development is primarily regulated by water availability during early and mid-growth stages, with irrigation exerting consistent effects at bud initiation, vegetative growth, and flowering. This sensitivity reflects the strong influence of soil moisture on early shoot elongation and internode expansion in C_3_ crops (Chaves et al., 2009; Razzaghi et al., 2012), and year-to-year differences likely reflect greater phenological resolution of plant height measurements in 2025 than in 2024. The absence of significant irrigation × nitrogen interactions, together with convergence in plant height at maturity, suggests that water and nitrogen effects on canopy architecture are largely additive and that final plant stature is buffered against early-season variability in resource availability (Hinojosa et al., 2018).

Grain yield responses indicate that nitrogen availability was the dominant driver of quinoa productivity under humid temperate conditions, while the absence of a significant irrigation main effect or irrigation × nitrogen interaction demonstrates strong yield resilience to moderate reductions in water supply. This resilience is consistent with previous studies reporting quinoa’s tolerance to limited water availability and its capacity to maintain yield under moderate deficit conditions (Razzaghi et al., 2012; Hinojosa et al., 2019). Although the high-yielding genotype consistently produced greater absolute grain yield than the low-yielding genotype across all irrigation and nitrogen treatments, both genotypes exhibited similar physiological, canopy, and yield response patterns to management, as reflected by the absence of significant genotype × irrigation or genotype × nitrogen interactions. This indicates that differences in yield potential were primarily attributable to inherent genotypic capacity rather than differential sensitivity to water or nitrogen supply. The consistent yield response to nitrogen across irrigation regimes and genotypes further supports the interpretation that improved agronomic efficiency of nitrogen, rather than increased nitrogen input or irrigation intensity, governed yield formation in this study.

## 5 Conclusions

This study demonstrates that physiological performance, canopy development, grain yield, and agronomic efficiency of nitrogen (AE_n_) in quinoa are regulated by irrigation and nitrogen supply in a growth-stage–dependent manner under humid temperate conditions in the U.S. Midwest. Irrigation primarily constrained stomatal conductance and leaf-level gas exchange during early development and flowering, whereas nitrogen availability exerted the strongest influence during grain filling, identifying post-anthesis development as a primary physiological control point for yield formation and nitrogen efficiency. Although nitrogen application enhanced photosynthetic capacity and canopy vigor, further increases in nitrogen did not yield proportional gains in yield, resulting in reduced AE_n_ and increased disease risk at higher nitrogen rates. Grain yield was resilient to moderate late-season reductions in supplemental irrigation, and an intermediate nitrogen rate (75 kg N ha^−1^) was sufficient to achieve maximum productivity across irrigation regimes, indicating additive rather than interactive effects of irrigation and nitrogen. Although the high-yielding genotype produced greater absolute grain yield, both genotypes exhibited similar physiological, canopy, yield, and AE_n_ responses to irrigation and nitrogen management, indicating limited genotype × management interactions.

Future research should quantify stage-specific irrigation thresholds in controlled and semi-controlled environments (e.g., rain-out shelters or high tunnels) to disentangle rainfall effects in humid temperate systems and to evaluate refined nitrogen timing strategies that better synchronize nitrogen availability with grain filling. Integrating physiological measurements with biochemical indicators of nitrogen assimilation (e.g., nitrate reductase activity) and high-throughput phenotyping tools, such as UAV-based vegetation indices, will further improve mechanistic understanding and support precision irrigation and nitrogen management strategies for quinoa in non-traditional production environments.

## Data Availability

The original contributions presented in the study are included in the article/**Supplementary Material**. Further inquiries can be directed to the corresponding author.
