# Supplementary material for: Physiological, canopy, and yield responses of quinoa to irrigation and nitrogen management in the U.S. Midwest
Source: Front Plant Sci. 2026 Mar 12;17:1780275. doi: 10.3389/fpls.2026.1780275 (PMC13019640; doi:10.3389/fpls.2026.1780275)
Supplement: Supplementary file 1 [file Table1.docx]

**Table S1**. Analysis of variance (ANOVA) results for the effects of variety, irrigation, nitrogen fertilizer rate, and their interactions on normalized difference vegetation index (NDVI) across days after planting (DAP). Values represent P-values for main effects and interactions evaluated at 15, 22, 34, 40, 48, 62, 78, and 89 DAP. Significance levels are indicated as P < 0.05, P < 0.01, and P < 0.001.

| Source | 15DAP | 22DAP | 34DAP | 40DAP | 48DAP | 62DAP | 78DAP | 89DAP |
| --- | --- | --- | --- | --- | --- | --- | --- | --- |
| Var | 0.7726 | 0.2415 | 0.0138 | 0.0998 | 0.6957 | <.0001 | 0.0001 | <.0001 |
| Irr | 0.0001 | 0.014 | 0.0354 | 0.047 | 0.0001 | 0.0109 | 0.0001 | <.0001 |
| Var x Irr | 0.1816 | 0.039 | 0.0001 | 0.0001 | 0.0151 | 0.2419 | 0.0733 | 0.0847 |
| Nitro. | 0.956 | 0.7069 | 0.0429 | .0001 | 0.0001 | 0.0801 | 0.0095 | <.0001 |
| Var x Nitro. | 0.8289 | 0.9213 | 0.92 | 0.7649 | 0.7895 | 0.3687 | 0.5012 | 0.1216 |
| Irr x Nitro. | 0.9671 | 0.9501 | 0.85 | 0.5091 | 0.2598 | 0.7488 | 0.7027 | 0.7563 |
| Var x Irr x Nitro. | 0.9515 | 0.8929 | 0.7683 | 0.8022 | 0.8585 | 0.4667 | 0.443 | 0.3207 |

*, **, *** significant differences at *p* <0.05, *p* <0.01, and *p* <0.001, respectively.

**Table S2.** Analysis of variance (ANOVA) showing the effects of irrigation, nitrogen, variety, and their interactions on quinoa grain yield. The significance levels are indicated as P < 0.05, P < 0.01, P<0.001

| Source of Variation | df | F-value | p-value | Significance |
| --- | --- | --- | --- | --- |
| Irrigation | 3 | 1.81 | 0.158 | ns |
| Nitro | 2 | 11.82 | 0.001 | ** |
| Var | 1 | 4.65 | 0.036 | * |
| Irr × Nitro | 6 | 0.76 | 0.608 | ns |
| Irr × Var | 3 | 0.79 | 0.508 | ns |
| Var x Nitro | 2 | 0.13 | 0.883 | ns |
| Var x Irr × Nitro | 6 | 0.66 | 0.684 | ns |

*, **, *** significant differences at p <0.05, p <0.01, and p <0.001, respectively.

**Table S3**. Analysis of variance (ANOVA) for agronomic efficiency of nitrogen (AEₙ) showing the effects of genotype (Var), nitrogen rate (Nitro), irrigation regime (Irr), and their interactions. Significance levels are indicated as P < 0.05 (*), P < 0.01 (**)

| Source | Nparm | DF | F Ratio | Prob > F | Significance |
| --- | --- | --- | --- | --- | --- |
| Var | 1 | 1 | 0.4091 | 0.5273 | ns |
| Nitro | 1 | 1 | 1.0144 | 0.3219 | ns |
| Irr | 3 | 3 | 2.9321 | 0.0495 | * |
| Var x Nitro | 1 | 1 | 0.2701 | 0.6071 | ns |
| Var x Irr | 3 | 3 | 1.5687 | 0.2175 | ns |
| Irr x Nitro | 3 | 3 | 0.7156 | 0.5504 | ns |
| Var × Irr × Nitro. | 3 | 3 | 0.213 | 0.8866 | ns |

*, **, *** significant differences at *p* <0.05, *p* <0.01, and *p* <0.001, respectively.

**Table S4.** Pre-plant soil chemical properties at the experimental site. Soil samples were collected before nitrogen fertilizer application from two depths (0–20 cm and 20–40 cm). Values represent baseline soil fertility and inorganic nitrogen status used to contextualize nitrogen and irrigation responses.

| Sample ID | Depth (cm) | pH | OM (%) | CEC (meq 100 g⁻¹) | NO₃⁻-N (ppm) | NH₄⁺-N (ppm) | TKN (mg kg⁻¹) | Organic N (mg kg⁻¹) | Bray-P (lb A⁻¹) | Ca (lb A⁻¹) | Mg (lb A⁻¹) | K (lb A⁻¹) |
| --- | --- | --- | --- | --- | --- | --- | --- | --- | --- | --- | --- | --- |
| Depth 1 | 0–20 | 5.6 | 0.8 | 11.2 | 2.02 | 9.67 | 1036 | 1025 | 75 | 2898 | 396 | 236 |
| Depth 2 | 20–40 | 5.8 | 1.2 | 9.6 | 1.49 | 5.15 | 1177 | 1170 | 76 | 2342 | 362 | 207 |

**Table S5.** Realized irrigation, rainfall, and total water input (Rain + Irrigation) for quinoa irrigation treatments in 2024 and 2025. Seasonal irrigation treatments were defined using an ET₀-based supplemental irrigation target of 430 mm, which established the full-irrigation daily rate (4.26 mm d⁻¹) and proportional deficit treatments (PI = 75%, DI = 50%, EDI = 25%) reported in Table 1. All plots received identical full irrigation during an early-season phase; differential irrigation treatments were imposed only during a late-season treatment window. Rainfall was calculated from daily observations in the on-site weather dataset and summed over the irrigation-managed period. Differences in total water input among treatments, therefore, reflect treatment-specific irrigation rates during the late-season window.

| Year | Treatment | Phase 1 irrigation (mm) | Phase 2 irrigation (mm) | Total irrigation applied (mm) | Rainfall (mm) before irrigation treatment | Rainfall (mm) after irrigation treatment | Total Rainfall (mm) | Total water input (mm) |
| --- | --- | --- | --- | --- | --- | --- | --- | --- |
| 2024 | Full | 178.92 | 174.66 | 353.58 | 233.17 | 105.16 | 338.33 | 691.91 |
|  | PI (75%) | 178.92 | 130.79 | 309.71 | 233.17 | 105.16 | 338.33 | 648.04 |
|  | DI (50%) | 178.92 | 87.33 | 266.25 | 233.17 | 105.16 | 338.33 | 604.58 |
|  | EDI (25%) | 178.92 | 43.46 | 222.38 | 233.17 | 105.16 | 338.33 | 560.71 |
| 2025 | Full | 230.04 | 166.14 | 396.18 | 190.25 | 46.48 | 236.73 | 632.91 |
|  | PI (75%) | 230.04 | 124.41 | 354.45 | 190.25 | 46.48 | 236.73 | 591.18 |
|  | DI (50%) | 230.04 | 83.07 | 313.11 | 190.25 | 46.48 | 236.73 | 549.84 |
|  | EDI (25%) | 230.04 | 41.34 | 271.38 | 190.25 | 46.48 | 236.73 | 508.11 |

NB: Phase 1 is pre-irrigation treatment, and Phase 2 is after irrigation treatments start.

**Table S6:** Least-squares means (LSMeans ± SE) for stomatal conductance (gsw), transpiration (E), and intercellular CO₂ concentration (Ci) at booting, flowering, and grain-filling stages under the Variety × Irrigation interaction. Different superscript letters within each stage and trait indicate significant differences (Tukey HSD, P ≤ 0.05).

| Stage | Trait | Variety | Full | PI | DI | EDI |
| --- | --- | --- | --- | --- | --- | --- |
| Booting | gsw | Var1 | 2.02 ± 0.251^a^ | 1.16 ± 0.251^bcd^ | 0.66 ± 0.251^d^ | 0.76 ± 0.251^cd^ |
| Booting | gsw | Var2 | 1.37 ± 0.251^abcd^ | 1.68 ± 0.251^ab^ | 1.19 ± 0.251^bcd^ | 1.41 ± 0.251^abc^ |
| Booting | E | Var1 | 0.02 ± 0.001^a^ | 0.02 ± 0.001^ab^ | 0.01 ± 0.001^c^ | 0.02 ± 0.001^bc^ |
| Booting | E | Var2 | 0.02 ± 0.001^ab^ | 0.02 ± 0.001^a^ | 0.02 ± 0.001^a^ | 0.02 ± 0.001^a^ |
| Booting | Ci | Var1 | 292.23 ± 5.426^a^ | 288.42 ± 5.426^ab^ | 261.06 ± 5.426^c^ | 275.18 ± 5.426^bc^ |
| Booting | Ci | Var2 | 292.59 ± 5.426^a^ | 292.43 ± 5.426^a^ | 290.46 ± 5.426^ab^ | 298.16 ± 5.426^a^ |
| Flowering | gsw | Var1 | 0.33 ± 0.184^d^ | 0.40 ± 0.184^cd^ | 0.96 ± 0.184^ab^ | 0.69 ± 0.184^abcd^ |
| Flowering | gsw | Var2 | 0.59 ± 0.184^bcd^ | 0.89 ± 0.184^abc^ | 1.18 ± 0.184^a^ | 0.90 ± 0.184^abc^ |
| Grain filling | E | Var1 | 0.01 ± 0.001^c^ | 0.01 ± 0.001^bc^ | 0.01 ± 0.001^abc^ | 0.01 ± 0.001^bc^ |
| Grain filling | E | Var2 | 0.01 ± 0.001^bc^ | 0.01 ± 0.001^ab^ | 0.01 ± 0.001^abc^ | 0.01 ± 0.001^a^ |
| Grain filling | Ci | Var1 | 188.99 ± 20.039^ab^ | 178.42 ± 20.039^ab^ | 185.59 ± 20.039^ab^ | 137.48 ± 20.039^b^ |
| Grain filling | Ci | Var2 | 154.93 ± 20.039^ab^ | 189.13 ± 20.039^ab^ | 195.17 ± 20.039^a^ | 205.46 ± 20.039^a^ |

V1**,** PI698769 (origin: New Mexico, USA), V2, PI614885 (origin: Chile)

# **Table S7**. Interaction effects of irrigation regime and nitrogen rate on stomatal conductance (gsw), transpiration (E), and net photosynthetic rate (A) at the grain-filling stage. Values are means ± SE; different letters indicate significant differences among irrigation × nitrogen combinations (P < 0.05).

| Irrigation | N rate (kg ha⁻¹) | gsw (mol m⁻² s⁻¹) | E (mol m⁻² s⁻¹) | A (µmol m⁻² s⁻¹) |
| --- | --- | --- | --- | --- |
| Full | 0 | 0.303 ± 0.10^bcde^ | 0.010 ± 0.001^bcde^ | 18.751 ± 2.0^cd^ |
| Full | 75 | 0.355 ± 0.10^abc^ | 0.011 ± 0.001^abcd^ | 22.632 ± 2.0^bcd^ |
| Full | 150 | 0.159 ± 0.10^e^ | 0.006 ± 0.001^f^ | 18.812 ± 2.0^cd^ |
| PI | 0 | 0.211 ± 0.10^cde^ | 0.008 ± 0.001^def^ | 21.759 ± 2.0^bcd^ |
| PI | 75 | 0.358 ± 0.10^ab^ | 0.012 ± 0.001^abc^ | 25.392 ± 2.0^abcd^ |
| PI | 150 | 0.355 ± 0.10^abc^ | 0.012 ± 0.001^ab^ | 27.931 ± 2.0^abc^ |
| DI | 0 | 0.184 ± 0.10^e^ | 0.008 ± 0.001^ef^ | 17.084 ± 2.0^d^ |
| DI | 75 | 0.334 ± 0.10^bcd^ | 0.011 ± 0.001^abcd^ | 29.227 ± 2.0^ab^ |
| DI | 150 | 0.484 ± 0.10^a^ | 0.014 ± 0.001^a^ | 33.829 ± 2.0^a^ |
| EDI | 0 | 0.207 ± 0.10^de^ | 0.009 ± 0.001^cdef^ | 21.403 ± 2.0^bcd^ |
| EDI | 75 | 0.334 ± 0.10^bcd^ | 0.012 ± 0.001^abc^ | 28.192 ± 2.0^abc^ |
| EDI | 150 | 0.421 ± 0.10^ab^ | 0.013 ± 0.001^ab^ | 34.753 ± 2.0^a^ |

**Supplementary Methods S1. Reference Evapotranspiration (ETo) Calculation**

Daily reference evapotranspiration (ETo) was calculated using the FAO-56 Penman–Monteith method (Allen et al., 1998), which estimates ETo for a hypothetical, well-watered grass reference surface and is widely applied in crop water-use studies.

ETo was computed as:

ETo = [0.408 Δ (Rn − G) + γ (900 / (T + 273)) u₂ (es − ea)] / [Δ + γ (1 + 0.34 u₂)]

where ETo is reference evapotranspiration (mm day⁻¹), Rn is net radiation (MJ m⁻² day⁻¹), G is soil heat flux (MJ m⁻² day⁻¹), T is mean daily air temperature at 2 m height (°C), u₂ is wind speed at 2 m height (m s⁻¹), es is saturation vapor pressure (kPa), ea is actual vapor pressure (kPa), Δ is the slope of the saturation vapor pressure–temperature curve (kPa °C⁻¹), and γ is the psychrometric constant (kPa °C⁻¹).

Saturation vapor pressure was calculated from daily maximum and minimum air temperatures, and net radiation was estimated following FAO-56 procedures. For daily time steps, soil heat flux (G) was assumed to be zero. Wind speed measurements were standardized to a height of 2 m. Meteorological data were obtained from the USDA-NRCS Soil Climate Analysis Network (SCAN) weather station located at the George Washington Carver Farm (Site 2223), Jefferson City, Missouri, USA.
